# Connecting the dots: Examining stressful life events, campus climate, and school engagement on academic achievement and psychological distress in a predominantly Latine sample

**DOI:** 10.1111/jora.70096

**Published:** 2025-11-24

**Authors:** Ilene N. Cruz, Kyle A. Moreno, Brittany Stovall, Luis Paz de la Vega, Gabriela Chavira

**Affiliations:** ^1^ Department of Human Development and Family Sciences University of Texas at Austin Austin Texas USA; ^2^ Rossier School of Education University of Southern California Los Angeles California USA; ^3^ Department of Psychology California State University Northridge California USA; ^4^ Department of Psychology Los Angeles Valley College Los Angeles California USA

**Keywords:** academic achievement, campus climate, high school students, historically marginalized adolescents, psychological distress, school engagement, stressful life events

## Abstract

Historically marginalized adolescents experience environmental stressors that contribute to psychological distress and compromised academic outcomes. Prior research highlights the role of a positive school climate and school engagement in promoting both academic achievement and psychological well‐being. Building on this work, we propose school engagement may mediate relationships between stressful life events (SLEs) and campus climate on academic achievement and psychological distress. In a sample of 293 historically marginalized adolescents (81.2% Latine), path analyses showed mediation between campus climate, SLEs, and psychological distress via school engagement. Additionally, school engagement partially mediated the link between SLEs and academic achievement. Multiple regression analyses revealed school‐related challenges were associated with lower school engagement, more psychological distress, and lower academic achievement. These findings highlight the critical role of school engagement in shaping both academic and psychological outcomes. Implications for equity‐focused initiatives are discussed.

Since the 1990s, suburban areas in the United States have undergone significant demographic transformations, with historically marginalized groups moving into neighborhoods once dominated by White, middle‐ and upper‐middle‐class populations (Frey, 2022). Resultantly, suburbs in major metropolitan areas are now more racially diverse than the nation overall, introducing new structural and institutional complexities for suburban schools (Frankenberg & Siegel‐Hawley, 2024; Lewis‐McCoy, 2014). In response to demographic shifts, suburban schools are increasingly serving a more racially and socioeconomically diverse student population. In parallel, educational attainment in the United States has risen substantially in recent decades, with the national high school graduation rate reaching 87% in 2021–2022 (National Center for Education Statistics [NCES], 2024).

In this evolving demographic context, nurturing productive and thriving transitions from adolescence to adulthood is critical for cultivating psychological resilience and academic success (United Nations Children's Fund [UNICEF], 2022). Yet, adolescence is marked by heightened vulnerability to stressors (Kaczmarek & Trambacz‐Oleszak, 2021), including stressful life events (SLEs), such as violence, grief, and family instability (Dohrenwend, 2000). Dohrenwend (2000) explains SLEs are occurrences that disrupt an adolescent's routine and demand psychological and behavioral readjustment.

In addition to understanding the pernicious effects of SLEs, prior literature has well‐documented how violence and other SLEs disproportionately impact ethnically racially marginalized and impoverished adolescents (Santacrose et al., 2021; Whipple et al., 2021). Latine and Black adolescents, in particular, experience higher rates of exposure to violence compared with their White peers (Crouch et al., 2000). Such disparities are deeply rooted in systemic inequities tied to neighborhood disadvantage, unequal access to education, and historical patterns of segregation (Armstead et al., 2019). Among these challenges, schools may serve as sanctuaries, offering consistent structure, socioemotional support, and a sense of belonging (Nickerson et al., 2021). As such, teachers and school staff play a vital role in mitigating the effects of adversity by fostering caring relationships and inclusive environments (Gregory et al., 2010; Lenzi et al., 2017).

Understanding how adolescents respond to SLEs within school settings is essential in an increasingly diverse society. This study draws on the Integrative Model for the Study of Developmental Competencies in Minority Children (García Coll et al., 1996), which emphasizes how social position factors (e.g., race, class) shape developmental outcomes through their influence on proximal developments (e.g., schools). Within this framework, school engagement is positioned as a promotive mechanism that can bridge the effects of both supportive (e.g., campus climate) and adverse (e.g., SLEs) conditions on academic and psychological outcomes. Specifically, we examine how school engagement mediates the relationships between campus climate, SLEs, academic achievement, and psychological distress in a sample of historically marginalized adolescents attending a suburban high school in California, United States.

## Theoretical framework

The study is grounded in the Integrative Model for the Study of Developmental Competencies in Minority Children (García Coll et al., 1996), which emphasizes the role of social position factors (e.g., race‐ethnicity, socioeconomic status) in shaping developmental outcomes through their cumulative impact on proximal environments (i.e., school, family, and neighborhood). According to this model, these social position factors operate through mechanisms of social stratification (e.g., discrimination, segregation, and limited access to resources), which in turn shape the quality of developmental contexts. Among the various developmental contexts (e.g., neighborhood) emphasized in the model, schools represent a critical setting during adolescence. Specifically, school‐situated promotive and inhibiting environments (e.g., campus climate) are related to adolescents' school engagement, in tandem with individual characteristics (e.g., gender) and familial characteristics (e.g., socioeconomic status, generation status), to directly shape developmental competencies. Originally developed with urban, historically marginalized adolescents, the model has since been extended to immigrant and rural contexts (Stein et al., 2016). While the model functions similarly across urban and rural settings, our findings from a suburban context suggest additional theoretical refinements that may enhance its applicability across diverse settings.

Relevant to the current study, school engagement is conceptualized as a promotive mechanism that emerges in response to the quality of adolescents' school environments. Relatedly, one key dimension of school engagement is campus climate. From a systems view of school climate perspective (Rudasill et al., 2018), school engagement is defined as the collective perceptions of students, teachers, and school personnel regarding the quality of social interactions, relationships, safety, and shared values within a school. A positive campus climate, in turn, may foster school engagement by providing historically marginalized adolescents with a sense of belonging, motivation, self‐efficacy, and academic purpose. Conversely, SLEs, such as exposure to violence, family instability, or academic struggles, represent inhibiting environmental conditions that may disrupt adolescents' ability to engage in school and maintain psychological well‐being. In this study, we extend the model by proposing that school engagement functions as a key promotive mechanism that mediates the effects of both promotive (e.g., campus climate) and inhibiting (e.g., SLEs) environmental factors on academic achievement and psychological distress. This framing allows us to examine how school engagement may explain the link between developmental contexts and adolescent outcomes for historically marginalized adolescents navigating stratified educational contexts.

A core tenet of the integrative model is its recognition of culturally grounded competencies (i.e., cognitive, social, emotional, linguistic, biculturalism, coping with racism) historically marginalized adolescents bring to the ecologies they inhabit. Building on this framework, we propose two additional competencies that are highly salient in today's evolving educational and psychological demands: academic competency and coping with stress. Because educational credentials have increasingly structured access to higher education and stable employment (Carnevale et al., 2023), academic skills function less as optional assets and more as essential developmental competencies. Academic competency, while traditionally considered an outcome of other developmental processes (e.g., cognitive, linguistic), also functions as a dynamic and culturally mediated skillset shaped by adolescents' efforts to navigate stratified school environments (Quiroga et al., 2013; Zaff et al., 2017). Similarly, coping with stress—distinct from coping with racism—captures the psychological strategies historically marginalized adolescents use to manage chronic and cumulative stressors related to academic performance, exposure to violence, familial loss, and other life‐altering transitions (Compas et al., 2001; Sheffler et al., 2020). Thus, incorporating these competencies expands its relevance to suburban contexts and reflects the layered developmental demands facing historically marginalized adolescents.

## Stressful life events and psychological adjustment in school

National studies show that ~ 8.5% of adolescents are exposed to community violence (Ullmann et al., 2021), 30.2% experience familial loss (Aldridge & McChesney, 2018), and 27.5% experience family structure‐altering transitions (Anderson et al., 2014). However, historically marginalized adolescents, such as Black or Latine, are more likely to experience more SLEs than their White peers (Martin‐Gutierrez et al., 2021). These events can disrupt emotional, neurobiological, psychological, and physical development, leading to academic difficulties, mental health challenges, and increased risk of negative life outcomes (Losen & Martinez, 2013; National Institute of Justice, 2016; Smith & Pollak, 2020).

Chronic exposure to SLEs in adolescence is associated with depression (LeMoult et al., 2020; Pine et al., 2002), anxiety (Juruena et al., 2020), and suicidality (Yıldız, 2020). These stressors contribute to school‐related challenges, such as disengagement and pushout (Dupéré et al., 2018; Ouyang et al., 2021). However, the consequences of SLE exposure for psychological functioning may vary by gender. For example, girls are more likely to experience internalizing symptoms (e.g., anxiety), whereas boys are more likely to exhibit externalizing symptoms (e.g., physical aggression; Rosenfield & Mouzon, 2013). Furthermore, while adolescent girls and boys report similar levels of SLEs, girls tend to experience interpersonal‐related (e.g., relationships) stressors, while boys tend to face school‐ or authority‐related (e.g., teachers, police) stressors, leading to higher school pushout risk among boys (Lavoie et al., 2019). In addition, for adolescents from low‐income and historically marginalized groups, the impact of SLEs is compounded by chronic community violence and the cumulative stress of living in under‐resourced environments, which elevate stress, impair academic performance, and increase vulnerability to psychological illnesses, including depression (Brondolo et al., 2018; Goodman et al., 2005; Lepore & Kliewer, 2013). Given these patterns and the central role schools play in adolescents' lives, school‐related challenges (i.e., a type of SLE) are likely to be particularly salient during this developmental period. Accordingly, we also explored the unique associations between school‐related challenges on school engagement, psychological distress, and academic achievement in the present study.

## The association between campus climate and academic achievement

Campus climate refers to the collective experiences of school life, encompassing norms, values, goals, relationships, and organizational structures that shape student development (Thapa et al., 2013; Wang & Degol, 2016). For instance, a positive campus climate includes high‐quality teaching, effective learning practices, an inclusive school culture, and engagement from teachers, parents, and peers (Cohen et al., 2009; Rudasill et al., 2018). At the school level, it is further characterized by innovation, adaptability, high morale, and effective communication (Rudasill et al., 2018). Similar work by Wang and Degol (2016) identifies safety, community, academic environment, and institutional support as key dimensions of campus climate.

Research shows that a positive campus climate fosters a sense of safety and belonging among adolescents (Kutsyuruba et al., 2015). For example, among Californian adolescents aged 10–18, a stronger sense of community and greater teacher support were associated with increased feelings of safety at school (Lenzi et al., 2017). Similarly, Gregory et al. (2010) found that Virginian adolescents with supportive and caring adults in their schools experienced less bullying and greater emotional security. These findings suggest that campus climate may influence both academic success and psychological distress, particularly for adolescents facing SLEs. Central to the benefits of a positive campus climate are the interpersonal relationships that exist within it. Constructs such as school connectedness, cultural inclusion, and community engagement play a vital role in nurturing these relationships (Bradshaw et al., 2014; Darling‐Hammond & Cook‐Harvey, 2018). In addition, respect for diverse backgrounds and active family involvement further strengthen students' academic and emotional outcomes (Banerjee et al., 2011).

At the classroom level, respectful and inclusive communities enhance students' learning experiences (Wong et al., 2021). Teaching strategies, such as clear expectations, meaningful content, and supportive feedback, promote student engagement and self‐efficacy (Wong et al., 2021). These practices deepen students' emotional and mental investment in learning and reduce disengagement. In turn, a positive classroom climate is associated with lower levels of substance use, absenteeism, and mental health concerns (Pietarinen et al., 2014; Thapa et al., 2013). Likewise, a positive classroom climate has been shown to boost adolescents' mental focus and resilience, safeguarding against burnout and other adverse outcomes (Furrer et al., 2014; Pietarinen et al., 2014).

Conversely, when campus climate is marked by racial discrimination or exclusion, it may erode students' sense of belonging and hinder academic and emotional development (Thapa et al., 2013). Thus, cultivating a positive campus climate is essential for promoting academic achievement and well‐being, as well as mitigating the harmful effects of unsupportive proximal environments. Collectively, this research indicates that campus climate may serve as a promotive or inhibitive environment. As such, we examine whether campus climate is associated with academic achievement (i.e., a product of academic competency) among historically marginalized adolescents attending suburban schools.

## The association between school engagement on achievement and psychological distress

School engagement encompasses students' interest in school, investment in their academic outcomes, and sense of connectedness to their school community (Dotterer & Lowe, 2011; Engels et al., 2016). Conceptualized as a multidimensional construct, school engagement encompasses behavioral (e.g., attendance, participation), emotional (e.g., sense of belonging), and cognitive (e.g., effort and persistence in learning) components (Fredricks et al., 2004). Extensive research links school engagement to positive outcomes, including academic achievement (Boutakidis et al., 2014; Plunkett et al., 2009), academic resilience (Finn & Rock, 1997), academic motivation (Wang & Eccles, 2013), psychological well‐being (Skinner et al., 2016; Upadyaya & Salmela‐Aro, 2013), and high school completion (Wang & Fredricks, 2013). Among Latine adolescents, higher engagement is associated with stronger academic performance throughout their educational trajectories (Boutakidis et al., 2014; Plunkett et al., 2009). Engaged students also tend to earn higher grades and perform better on standardized tests, while disengaged students are likely to struggle academically and exhibit problem behaviors, including school pushout (Gonzales et al., 2014; Piscitello et al., 2022; Wang & Fredricks, 2013). Emotional and cognitive engagement have also received empirical support for their utility relative to adolescents' depressive symptoms (Wang & Peck, 2013). Thus, the present study seeks to replicate scholarship (Boutakidis et al., 2014; Plunkett et al., 2009; Wang & Peck, 2013) between school engagement and academic achievement and psychological distress, but we also consider a broader range of promotive and inhibiting environments (i.e., campus climate and SLEs), and examine these links among adolescents living in suburban contexts.

## Purpose of study

Although prior research has documented individual associations among school climate, SLEs, psychological distress, and academic achievement, few studies have examined how these processes operate together, particularly through the lens of school engagement. To the authors' knowledge, few studies have examined school engagement as a mediator between campus climate, SLEs, and academic achievement. One study conducted among undergraduate college students in Ethiopia found that school engagement mediated the relation between campus climate and GPA (Berhanu & Sewagegn, 2024). Similarly, another study conducted among Black high school students in the United States found that behavioral and cognitive engagement mediated the link between racial fairness, a component of campus climate, and academic achievement (Griffin et al., 2017). A third study, conducted with junior high school students in China, also examined related constructs, identifying psychological capital and flexibility as mediators between SLEs and school engagement (Fang & Ding, 2023). As such, this is the first study to integrate SLEs, campus climate, and school engagement within a single model to examine their combined influence on both psychological distress and academic achievement. This gap is especially significant given the compounding impact of inhibiting/promotive contexts on historically marginalized adolescents in U.S. educational settings.

To investigate how schools may serve as a protective context amid the SLEs adolescents face, we examined the relationship between campus climate and school engagement in a sample of historically marginalized adolescents attending a suburban high school. Considering campus climate and SLEs are systemic factors outside adolescents' control, our investigation aims to elucidate the potential of school engagement in promoting academic achievement and mitigating psychological distress. Given the proposed relations among these types of promotive and inhibiting environments (García Coll et al., 1996), this study was guided by three central research questions: (1) To what extent does school engagement mediate the relationships between campus climate, SLEs, academic achievement, and psychological distress among historically marginalized adolescents? (2) What is the prevalence of SLEs among historically marginalized adolescents?, and (3) Which specific types of SLEs most strongly predict school engagement, academic achievement, and psychological distress among historically marginalized adolescents? Thus, we proposed the following hypotheses: (1) School engagement will mediate the relationship between campus climate and academic achievement, (2) School engagement will mediate the relationship between SLEs and academic achievement, (3) School engagement will mediate the relationship between SLEs and psychological distress, and (4) Among the types of SLEs, school‐related challenges will be the strongest negative predictor of school engagement, academic achievement, and psychological distress.

## METHODS

### Sample characteristics

The sample consisted of 293 ninth and tenth grade high school students with an average age of 14.82 years (*SD* = 0.88, range 14–19 years), with more (57%) ninth graders than tenth graders (43%). The sample demonstrated a balanced gender distribution, with 50.9% identifying as female and 49.1% identifying as male. The majority of adolescents (81.2%) identified as Latine, followed by multiethnic (9.2%), Black (5.1%), Asian (1.7%), Middle Eastern (1.4%), and Indigenous American (1.4%). Regarding birthplace, the majority of adolescents were U.S.‐born (85.9%). Among those born outside of the U.S. (14.1%), the average age of immigration to the U.S. was 5.06 years (*SD* = 3.74, range = 1 week to 16 years). In contrast to the adolescents, few mothers and fathers were U.S.‐born (20.5% and 21.7%, respectively), with the majority (42.4% and 41.3%) born in Mexico. The average level of education for mothers and fathers was 10.8 years (*SD* = 3.3) and 10.8 years (*SD* = 3.3), respectively. With regard to family income, parents reported a combined median income for family members ranging from $25,000 to $34,999.

### Procedure

The research study was approved by the university's institutional review board and the high school principal prior to commencing data collection. Participants were selectively recruited from ninth grade Life Skills courses and tenth grade English courses at a suburban high school located in Southern California, United States. The school was predominantly Latine (71.1%) and low‐income, with 66% of adolescents receiving free or reduced lunch at the time of data collection. The research team provided interested students with survey packets including parental consent forms (English/Spanish), adolescent assent forms (English/Spanish), a family information form, and a 10‐page survey, which took ~ 30 min to complete. Clear instructions were given for students to complete the survey packets at home and return them on the subsequent school day. All participants who returned completed and signed packets were given $10 as compensation for their participation. Further, to uphold confidentiality, all surveys were linked to consent forms via a three‐digit identification number. This coding system allowed the participants to be matched with their surveys, school record data, and census data.

### Measures

The demographic characteristics of the participants were evaluated using standard demographic items. Composite scores were computed for each scale by averaging the items, and reliability scores were determined using Cronbach's alpha to assess the internal consistency of the measures.

#### Campus climate (*α* = .91)

Campus Climate was a composite of two scales: the School Climate Survey and the Classroom Climate Scale.

##### School climate survey (*α* = .85)

Developed by Aber et al. (2000), school climate was measured using an adapted scale from the 24‐item School Climate Survey (High School Version). The survey was designed to assess various facets of school climate, including subscales of school fairness, student voice/participation, mutual respect among students, and social support from teachers. A sample item was, “In school we have a strong sense of responsibility to help and support one another.” The response choices varied along a four‐point Likert scale, with response choices ranging from 1 = *strongly disagree*, 2 = *somewhat disagree*, 3 = *somewhat agree*, and 4 = *strongly agree*. The scales were scored by calculating the average of each value, where a higher score indicated a more positive climate.

##### Classroom climate scale (*α* = .85)

Developed by Miller‐Johnson et al. (2004) for the Multisite Violence Prevention Project, the 18‐item Classroom Climate Scale was adopted in the current study to measure three components of students' perceptions of their classroom climate: student–student relationships, student–teacher relationships, and awareness/reporting. Respondents indicated the extent to which they agree or disagree with a series of declarative statements. A sample item was, “Teachers treat students with respect.” Each subscale ranged on a four‐point Likert scale, with response choices ranging from 1 = *strongly disagree*, 2 = *somewhat disagree*, 3 = *somewhat agree*, and 4 = *strongly agree*. Scales were scored by calculating the average of each value, where a higher score indicated a more positive climate.

#### Stressful life events

The Stressful Urban Life Events Scale (Attar et al., 1994) measured participants' stressors over the past year, sourced from the compendium of assessment tools published by the Centers for Disease Control and Prevention (Dahlberg et al., 2005). This 15‐item checklist of independent life events comprised four subdomains measuring exposure to violence (five items), familial loss (three items), school‐related challenges (three items), and family structure‐altering transitions (four items). Given that the checklist of life events in each subscale is not dependent upon each other, internal consistency was not appropriate to compute. For example, while events such as, “Did you change where you went to school?” and “Has a new baby come into the family?” (family structure‐altering transitions) may both elicit stress, they are not necessarily interrelated. Other sample items included, “Have you seen or been around people shooting guns?” (exposure to violence), “Did a family member die?” (familial loss), and “Did you get poor grades on your report card?” (school‐related challenges). Participants responded with 0 for *no* and 1 for *yes*. A total score was calculated by summing the number of events with higher scores indicating greater exposure to stressors. Subdomain scores were calculated the same way, with higher scores indicating greater exposure to specific stressor types.

#### School engagement (*α* = .81)

School engagement was a composite of two scales: the Commitment to School Scale and the Attitudes Toward School Scale.

##### Commitment to school scale (*α* = .76)

Originally developed and validated across Black, Asian, White, Latine, and Indigenous American ethnic‐racial groups by Glaser et al. (2005), the six‐item Commitment to School Scale measured students' feelings about the importance of school and coursework. A sample item included, “How often do you feel that the schoolwork you are assigned is meaningful and important?” Each scale ranged on a five‐point Likert scale, with response choices ranging from 1 = *never*, 2 = *seldom*, 3 = *sometimes*, 4 = *often*, 5 = *almost always*. The scale was scored by calculating the average for each respondent with higher scores indicating stronger commitment to school.

##### Attitudes toward school scale (*α* = .56)

Developed by the Institute of Behavioral Science (1990), the five‐item Attitudes Toward School Scale measured students' feelings regarding various aspects of schooling, including homework and teachers' opinions. A sample statement included: “Education is so important that it's worth it to put up with things about school that I don't like.” Each scale ranged on a four‐point Likert scale, with response choices ranging from 1 = *strongly disagree*, 2 = *disagree*, 3 = *agree*, 4 = *strongly agree*. The scale was scored by calculating the average for each respondent with higher scores indicating more positive attitudes toward school.

#### Psychological distress (*α* = .83)

To assess psychological distress, the 12‐item Subjective Experience of Distress subscale from the Weinberger Adjustment Inventory (Weinberger & Schwartz, 1990) was utilized. Participants were instructed to select the response that best represented their current feelings. Sample statements included, “I feel nervous or afraid that things won't work out the way I would like them to,” and “I feel very unhappy.” Response options were: 1 = *never*, 2 = *not often*, 3 = *sometimes*, 4 = *often*, and 5 = *almost always*. Scores were computed by averaging responses across all items with higher scores indicating higher psychological distress. In a sample of Mexican adults, psychological distress yielded a Cronbach's alpha of *α* = .84 (Romo‐González et al., 2014).

#### Academic achievement (1 item)

To measure academic achievement, adolescents' grade point averages (GPA) were calculated using school transcripts for the fall and spring semesters of the academic year in which the data were collected. GPAs can range from 0 to 4.00, with higher levels indicating stronger academic performance. While GPA is an incomplete reflection of academic success, it continues to serve as the main academic benchmark for high school graduation and college admission, and was therefore selected as a key outcome in this study.

### Data analysis plan

**FIGURE 1:**
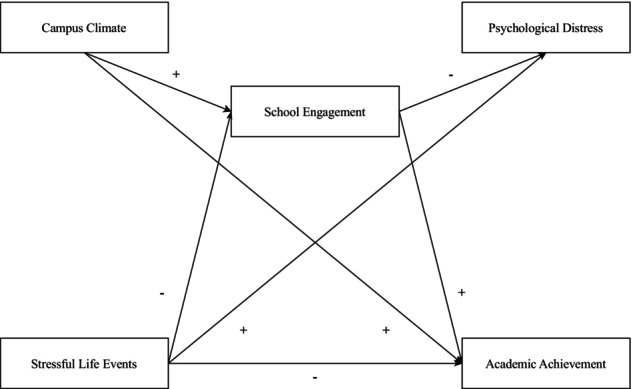
Hypothesized path model. + represents a hypothesized positive relationship, − represents a hypothesized negative relationship.

Data screening procedures were conducted using IBM SPSS version 29. This included evaluating missing values, multivariate outliers, multicollinearity, and adherence to participant criteria (e.g., ninth‐ and tenth‐grade students). Preliminary analyses involved conducting zero‐order correlations to explore the bivariate relationships among the variables. To address the first research question, a cross‐sectional path analysis was conducted in RStudio version 4.3.0 using the lavaan package, which uses Full Information Maximum Likelihood (FIML; Enders, 2010) to handle missing data (see Figure 1 for hypothesized model). Frequency analyses and multiple regression models, with a Bonferroni correction applied to control for Type I error, were also conducted in RStudio to address the second and third research questions. With five pairwise comparisons tested for each outcome, the corrected alpha level was .01 (i.e., .05 ÷ 5). Adolescents' self‐reported gender was added as a covariate.

### Model fit

Model fit was assessed using several indices, including Chi‐square goodness‐of‐fit (χ^2^), comparative fit index (CFI), root mean square error of approximation (RMSEA), and standardized root mean square residual (SRMR). A model is deemed to have a good fit if the χ^2^ is nonsignificant, CFI is greater than or equal to .95, RMSEA is less than or equal to .05, and SRMR is less than or equal to .08 (Kline, 2016).

## RESULTS

### Exploratory descriptives of stressful life events

The current sample revealed varying prevalence rates of SLEs. Notably, a substantial majority of the participants (95.2%) experienced at least one significant life stressor. Further analyses indicated that 89.1% encountered at least two events, while 75.4% experienced three or more SLEs. Additionally, specific stressor categories were examined, unveiling prevalent challenges in different domains: school‐related challenges were reported by 62.1% of adolescents, family structure‐altering transitions affected 66.9%, familial loss was experienced by 74.3%, and violence was witnessed by 61.4%.

### Pearson correlations

Pearson correlation analyses were conducted to examine and establish the strength and significance of the relationships between gender, school engagement, SLEs, campus climate, psychological distress, and academic achievement (see Table 1 for correlations, means, and standard deviations). Gender was positively associated with both psychological distress and academic achievement, suggesting that girls reported higher levels of each. School engagement was positively linked to academic achievement and negatively linked to psychological distress. SLEs were related to lower school engagement and academic achievement and higher psychological distress. Campus climate was strongly associated with greater school engagement and moderately linked to lower psychological distress. Notably, psychological distress was not directly associated with academic achievement.

**TABLE 1:** Pearson correlations, means, and standard deviations.

Variables	1	2	3	4	5	6
1. Gender^a^	–					
2. School engagement	.016	–				
3. Stressful life events	.067	−.211***	–			
4. Campus climate	−.006	.553***	−.160**	–		
5. Psychological distress	.149*	−.425***	.169**	−.297***	–	
6. Academic achievement	.139*	.315***	−.315***	.157**	−.102	–
Mean	0.510	3.281	0.315	4.169	2.184	2.144
SD	0.501	0.507	0.190	0.532	0.632	0.982

* *p* < .05.

** *p* < .01.

*** *p* < .001.

^a^ Gender was dummy coded as 0 = Boy and 1 = Girl.

### School engagement as a mediator

The current model fit the data well as indicated by the following fit indices: (χ^2^ [1, *N* = 293] = 1.712, *p* = .191, CFI = 0.997, TLI = 0.963, RMSEA = 0.049 [90% CI: 0.000, 0.173], SRMR = 0.011). As shown in Figure 1, we expected that school engagement would serve as a mediator in the relationship between campus climate and academic achievement. Specifically, we expected school engagement to partially explain the relationship between campus climate and academic achievement. Contrary to expectations, there was a nonsignificant negative direct effect between campus climate and academic achievement (*B* = −0.07, *β* = −.04, SE = 0.12, *p* = .567). However, campus climate had a significant positive indirect effect on academic achievement through school engagement (*B* = 0.27, *β* = .15, SE = 0.07, *p* < .001), indicating school engagement mediated the relationship between campus climate and academic achievement.

Second, we expected that school engagement would mediate the relationship between SLEs and academic achievement. As expected, there was a significant negative direct effect between SLEs and academic achievement (*B* = −1.42, *β* = −.27, SE = 0.28, *p* < .001). Moreover, SLEs had a significant negative indirect effect on academic achievement through school engagement (*B* = −0.18, *β* = −.04, SE = 0.08, *p* = .026), indicating that school engagement partially mediated the relationship between SLEs and academic achievement.

**FIGURE 2:**
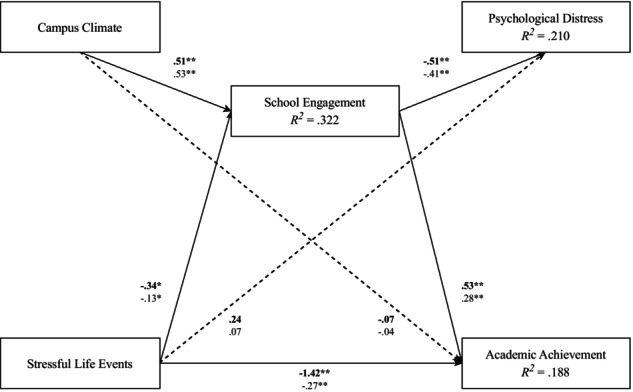
Final path model. Path model showing the relationship between the four study variables, mediated by school engagement, and controlling for gender. Bold text represents unstandardized coefficients. Regular text represents standardized coefficients. A solid line represents a significant relationship. A dotted line represents a nonsignificant relationship. The current model fit the data well: (χ^2^ [1, *N* = 293] = 1.712, *p* = .191, CFI = 0.997, TLI = 0.963, RMSEA = 0.049 [90% CI: 0.000, 0.173], SRMR = 0.011). *p < .01 **p < .001.

Third, we expected that school engagement would mediate the relationship between SLEs and psychological distress. Specifically, we expected school engagement to partially explain the relationship between SLEs and psychological distress. There was a nonsignificant positive direct effect between SLEs and psychological distress (*B* = 0.24, *β* = .07, SE = 0.18, *p* = .177). However, SLEs had a significant positive indirect effect on psychological distress through school engagement (*B* = 0.18, *β* = .05, SE = 0.07, *p* = .014), indicating school engagement mediated the relationship between SLEs and psychological distress (see Figure 2 for final model).

### Multiple regression analyses: School‐related challenges

A series of multiple regressions were conducted to investigate the four types of SLEs (school‐related challenges, family structure‐altering transitions, familial loss, and violence) and their impact on the outcomes (academic achievement and psychological distress) and the mediator (school engagement). In the first multiple regression, we examined the four types of SLEs as predictors of academic achievement and uncovered school‐related challenges (*B* = −1.43, *β* = −.49, SE = 0.16, *p* < .001) as a significant predictor, indicating that those with more school‐related challenges had lower levels of achievement.

In the second multiple regression, we examined the four types of SLEs as predictors of psychological distress and revealed school‐related challenges (*B* = 0.37, *β* = .20, SE = 0.11, *p* = .001) as a significant predictor, indicating that those with more school‐related challenges reported higher levels of psychological distress. In addition, gender emerged as a significant predictor of psychological distress (*B* = 0.21, *β* = .16, SE = 0.07, *p* = .005), indicating that adolescent girls perceived more psychological distress than adolescent boys.

In the final multiple regression, we examined the four types of SLEs as predictors of school engagement. The results revealed that both school‐related challenges (*B* = −0.69, *β* = −.45, SE = 0.08, *p* < .001) and familial loss (*B* = 0.24, *β* = .17, SE = 0.08, *p* = .003) were significant predictors of school engagement, indicating that those with more school‐related challenges reported lower levels of school engagement, while those who experienced more familial loss surprisingly reported higher engagement (see Table 2).

**TABLE 2:** Results of multiple regression analyses by stressful life event type.

Stressful life event	*t*	*p*	*β*	*F*	*df*	*p*	*R* ^2^
Academic achievement							
Overall model				20.415	5, 287	.001	.262
School‐related challenges	−1.431	.001*	−.485				
Family structure‐altering transitions	−0.140	.489	−.037				
Familial loss	−0.147	.339	−.052				
Violence	0.078	.716	.021				
Gender	0.180	.092	.092				
Psychological distress							
Overall model				4.126	5, 287	.001	.067
School‐related challenges	0.372	.001*	.196				
Family structure‐altering transitions	0.046	.752	.019				
Familial loss	0.080	.472	.044				
Violence	0.015	.921	.006				
Gender	0.207	.005*	.164				
School engagement							
Overall model				16.072	5, 287	.001	.219
School‐related challenges	−0.689	.001*	−.451				
Family structure‐altering transitions	−0.050	.639	−.026				
Familial loss	0.242	.003*	.166				
Violence	−0.086	.452	−.044				
Gender	−0.057	.296	−.056				

* *p* < .01.

## DISCUSSION

Guided by the Integrative Model (García Coll et al., 1996), which posits that the development of historically marginalized youth is shaped by their social position and the promoting/inhibiting environments they navigate, the study's findings offer theoretical support for conceptualizing academic competence and coping with stress as key developmental competencies. Under this framing, school climate, SLEs, and school engagement played critical environmental and relational contexts that either supported or hindered developmental outcomes. We discuss more specific findings below.

### Frequency of SLEs

Despite considerable variation in the types of SLEs, 95.2% of the sample reported experiencing at least one SLE in the past year. Among those who reported at least one SLE, 74.3% experienced familial loss, 66.9% experienced family structure‐altering transitions, 62.1% experienced school‐related challenges, and 61.4% witnessed violence in their community. Notably, these same stressors remained prevalent among those with more than one SLE (i.e., cumulative SLEs), with 40.9% reporting familial loss, 33.8% reporting family structure‐altering transitions, 30.7% reporting school‐related challenges, and 35.8% reporting violence exposure. By comparison, the prevalence of reported SLEs in the present study was substantially higher than those found in national studies surveying adolescents (Aldridge & McChesney, 2018; Anderson et al., 2014; Ullmann et al., 2021), potentially reflecting contextual differences in sample composition and geographic region. These findings contribute to the literature on historically marginalized adolescents by foregrounding the salience of SLEs in a suburban context, which remains underexplored relative to urban and rural populations.

### The mediating role of school engagement on academics

The study's findings may underscore the mediating role of school engagement in response to promotive factors (e.g., positive campus climate) and inhibiting stressors (e.g., SLEs) on adolescents' academic achievement and psychological distress. Notably, school engagement consistently functioned as a significant mediator: it linked perceptions of a positive campus climate to higher academic achievement; it linked more SLEs to greater psychological distress; and it partially accounted for the negative impact of SLEs on academic achievement. In each pathway, school engagement emerged as a conduit through which promotive and inhibiting factors may shape adolescent development.

That said, our first hypothesis was supported. While campus climate did not directly predict academic achievement, school engagement mediated this relationship. This suggests that a positive school environment alone may not be sufficient to improve academic outcomes, unless it also fosters student engagement. Other factors, such as aspirational capital, academic motivation, and parental involvement may also contribute to academic performance, particularly among historically marginalized adolescents who draw on cultural and familial capital to support their educational goals (Ayala & Contreras, 2018; Banerjee et al., 2011; Boutakidis & Rodriguez, 2022; Fan & Chen, 2001; Jeynes, 2016; Matos, 2015; Phinney et al., 2005). For example, adolescents may draw upon the cultural capital inherited through their families and communities and cultivate a distinct form of capital known as “finishing,” which serves as a catalyst in their academic aspirations (Matos, 2015).

More importantly, our results documented that school engagement acted as a pivotal intermediary mechanism between the campus atmosphere and adolescents' academic success (Matos, 2015). Prior empirical evidence suggests adolescents who actively participate in class, join campus clubs, engage in extracurricular sports, seek social support from teachers, and actively utilize the resources offered on campus are more likely to perform better academically (Bang et al., 2020; Chase et al., 2014; Garcia‐Reid et al., 2005). Additionally, adolescents who are actively engaged in school may develop higher levels of self‐efficacy and goal orientation, leading to greater academic success (Caraway et al., 2003).

Similarly, the results supported our second hypothesis of the mediating effect of school engagement between SLEs and academic achievement. Yet, the persistent negative impact of school‐related SLEs on academic achievement and psychological distress further illustrates how inhibiting environments, such as unsupportive school contexts, can disrupt developmental pathways. Specifically, the significant direct effect between SLEs and academic achievement suggests that the cumulative exposure to SLEs may impair adolescents' academic performance. It is possible that because SLEs are uncontrollable factors within adolescents' ecologies, high exposure to SLEs may predict poorer academic performance, subsequently overwhelming adolescents' ability to stay engaged in school (Qu et al., 2024; Stewart‐Tufescu et al., 2022). In addition, SLEs may affect academic achievement through mechanisms beyond school engagement, such as reduced cognitive capacity resulting from cumulative stress, which dysregulates the hypothalamic‐pituitary‐adrenal (HPA) axis and interferes with academic focus (Smith & Pollak, 2020). SLEs can also create external disruptions, such as financial instability or family upheaval, that contribute to ongoing stress and interfere with adolescents' academics (Liu & Merritt, 2018; Smith & Pollak, 2020).

### The mediating role of school engagement on psychological distress

The third hypothesis was supported, with school engagement emerging as a significant partial mediator between SLEs and psychological distress. Existing research has found that school engagement and social support from teachers and peers can buffer the negative effects of stress (Cohen & Wills, 1985; Rueger et al., 2016; Wang et al., 2018). Scholars have highlighted that adolescents experiencing elevated levels of psychological strain often struggle to stay engaged in school (Fröjd et al., 2008; McLeod et al., 2007), but a supportive school climate may help mitigate these effects. Therefore, schools that prioritize cultivating robust relationships could promote adolescents' resilience amidst adversity, including strengthening interpersonal connections, improving adolescents' perceptions of institutional trust and safety, enhancing adolescents' social and emotional skills, and minimizing punitive practices that may exacerbate trauma or stress (Murphey & Sacks, 2019).

### The role of school‐related challenges, gender, and familial loss

Supporting our fourth and final hypothesis, multiple regression analyses revealed that school‐related challenges were the most detrimental type of SLEs and were significantly associated with lower academic achievement, higher psychological distress, and reduced school engagement. School contexts may play a transformative role as proximal developmental environments that can either support or hinder marginalized adolescents' well‐being and academic trajectories. Because adolescents spend a substantial portion of their developmental time within school settings, school‐related stressors such as punitive discipline and experiences of racial marginalization may have a cumulative impact on psychological well‐being, disrupting both cognitive and emotional engagement (Losen & Martinez, 2013; Thapa et al., 2013; Wang & Peck, 2013). While this study did not examine school discipline or racial marginalization within the school's climate, we recommend for future research to examine these factors and their impact on student outcomes. Consequently, the lack of school engagement resulting from school‐related problems may increase adolescents' risk of high school pushout (Dupéré et al., 2018; Ouyang et al., 2021; Wang & Fredricks, 2013). These findings highlight the importance of transforming school environments to actively promote equity, inclusion, and psychological safety, consistent with the Integrative Model's call for systemic change to support the development of marginalized children (McChesney et al., 2015; Reyes et al., 2012).

Consistent with previous work, adolescent girls reported significantly higher levels of psychological distress compared with boys (Fröjd et al., 2008; Rosenfield & Mouzon, 2013). This finding aligns with scholarship suggesting that the type of stressors adolescents experience may differ by gender, with girls more likely to face interpersonal stressors and boys more likely to encounter authority‐ or school‐related stressors (Lavoie et al., 2019). Indeed, the gendered pattern of psychological distress observed may reflect culturally specific socialization processes that shape how adolescent girls experience and internalize stress. For example, Latine girls may be socialized to uphold marianismo, a traditional gender ideology that emphasizes self‐sacrifice, emotional strength, leadership, and caretaking responsibilities within family or peer networks. These cultural scripts may intensify the emotional weight of interpersonal stressors, particularly events involving family dynamics or relationship strain. Prior work has linked marianismo‐aligned gender norms to elevated internalizing symptoms, including depression and emotional distress (Nuñez et al., 2016). Within García Coll et al.'s (1996) Integrative Model, gender as a social position factor intersects with cultural norms to shape proximal environments, subsequently influencing both the types of stressors historically marginalized adolescent girls are exposed to and how they are emotionally processed.

Unexpectedly, we found that familial loss was associated with increased engagement in some cases, reflecting the Integrative Model's emphasis on adaptive culture, defined as the culturally grounded coping strategies and values that historically marginalized adolescents may draw upon to navigate adversity. When a supportive campus climate is nurturing and inclusive, these cultural strengths may be activated, helping adolescents remain engaged in school despite personal loss (Thapa et al., 2013; Wang & Degol, 2016). This form of cultural resilience aligns with prior research underlining how school engagement can promote psychological well‐being amid stress (Skinner et al., 2016). Thus, we highlight coping with stress as a developmental competency that may be shaped by adolescents' adaptive culture and supported through promotive school contexts. Finally, this finding may underscore the contextual and cultural specificity of developmental pathways emphasized in the Integrative Model's recognition of strengths‐based perspectives in the development of marginalized children (Aisenberg & Herrenkohl, 2008; García Coll et al., 1996; Li & Lerner, 2011; Ungar et al., 2007).

### Practical recommendations, future directions, and conclusions

Given our findings, we propose several implications that can better support historically marginalized adolescents exposed to SLEs in suburban school contexts. Central to these recommendations is the recognition that school engagement operates as a vital mechanism linking campus climate and SLEs to both academic achievement and psychological distress. As such, enhancing school engagement requires intentional, equity‐focused reforms that address systemic barriers.

First, schools might consider implementing culturally responsive and trauma‐informed practices that foster belonging, relevance, and emotional safety. Culturally relevant pedagogy (CRP) offers a foundational framework for affirming adolescents' cultural identities and fostering inclusive classroom environments (Ladson‐Billings, 1995). CRP has been shown to increase school engagement, academic achievement, and student‐teacher relationships, particularly for historically marginalized adolescents (e.g., Aronson & Laughter, 2016). When embedded into teacher preparation, CRP can help educators create more inclusive classroom environments that further reinforce these relational and academic benefits (Bottiani et al., 2018). At the same time, trauma‐informed approaches can be integrated into teacher professional development, enabling educators to recognize and respond to adolescents' stress‐related behaviors with empathy and structure. These approaches emphasize healing‐centered engagement, cultural responsiveness, and strategies to resist retraumatization (Palma et al., 2023). Given that exclusionary discipline practices, such as suspension and expulsion, can retraumatize youth and exacerbate disengagement, schools may benefit from adopting restorative practices that emphasize healing, accountability, and community‐building (Hemez et al., 2019; Lodi et al., 2021; Losen, 2015; Morgan, 2021). Specifically, these practices support adolescents' emotional regulation, strengthen relational trust, and cultivate safer, more inclusive learning spaces, offering them opportunities to build safe and caring relationships with educators in school (Brown et al., 2018; Sanders et al., 2023).

Second, fostering a positive campus climate requires strong partnerships between schools, families, and community organizations. Prior research indicates that parents from historically marginalized backgrounds are just as engaged, if not more engaged than their White counterparts, particularly among Latine families with lower parental educational attainment (Hanson et al., 2019). Schools can build on this strength by offering multilingual workshops, family support groups tailored to specific needs (e.g., single parents, recent immigrants), and consistent, affirming communication strategies to foster reciprocal partnerships with families and reinforce their ongoing contributions (Bryk et al., 2010; Gonzalez & Villalba, 2018; Martinez & Chavira, 2019; Yosso, 2005). Schools might also consider addressing the underrepresentation of teachers of color, a key factor shaping adolescents' sense of belonging and academic motivation (Carver‐Thomas, 2018). To illustrate, in the Los Angeles Unified School District (LAUSD)—the second largest school district in the United States—56.5% of high school students identify as Latine, compared with only 38.2% of high school teachers identifying as Latine (California Department of Education, 2025; LAUSD, 2023). Thus, recruiting and retaining diverse educators through supportive hiring practices, mentoring opportunities, and equitable workplace conditions can simultaneously improve school climate and academic outcomes (Bristol, 2020; Carver‐Thomas, 2018).

Lastly, this study underscores the importance of refining developmental models to better reflect the lived experiences of historically marginalized adolescents in suburban contexts. While the Integrative Model for the Study of Developmental Competencies in Minority Children (García Coll et al., 1996) provides a strong foundation, the inclusion of additional developmental competencies—coping with stress and academic competency—may enhance its applicability to youth navigating cumulative adversity as they continue to strive toward high school completion. Longitudinal moderation analyses are needed to examine how school engagement evolves over time and whether promotive environments, such as inclusive campus climates, provide sustained benefits in buffering against psychological distress. Specifically, future studies should explore how family‐level and school‐level variables interact to shape adolescents' academic trajectories amid cumulative SLEs. Mixed‐method approaches that amplify adolescents' voices can also provide deeper insight into the strategies youth use to navigate adversity and leverage school as a resource and refuge (Degal & Wang, 2016; Thapa et al., 2013). These potential findings are essential for informing more culturally and contextually responsive frameworks that extend beyond traditional urban or rural educational spaces. As the demographic landscape of American suburbs continues to shift, future research and policy must center the lived realities of historically marginalized adolescents to build equitable and trusting educational systems.

## AUTHOR CONTRIBUTIONS

**Ilene N. Cruz**: Conceptualization (lead); data curation (lead); formal analysis (lead); software (lead); visualization (lead); writing – original draft (equal); writing – review and editing (equal). **Kyle A. Moreno**: Conceptualization (equal); data curation (equal); formal analysis (equal); software (equal); validation (lead); visualization (equal); writing – original draft (equal); writing – review and editing (equal). **Brittany Stovall**: Writing – original draft (equal). **Luis Paz de la Vega**: Writing – original draft (equal). **Gabriela Chavira**: Conceptualization (equal); funding acquisition (lead); investigation (lead); supervision (lead); methodology (lead); writing – original draft (equal); writing – review and editing (equal).

## FUNDING INFORMATION

This research is supported by a subproject grant funded by the National Institute on Minority Health and Health Disparities (NIMHD) to the last author, grant number 1P20MD003938. The content is solely the authors' responsibility and does not represent the official views of the NIMHD.

## CONFLICT OF INTEREST STATEMENT

There is no conflict of interest for any of the study authors.

## ETHICS STATEMENT

All research related to human use complied with all relevant national regulations, institutional policies and the tenets of the Helsinki Declaration. The study was approved by the Institutional Review Board at California State University, Northridge, as well as the school principal at a charter high school in the Los Angeles area.

## PATIENT CONSENT STATEMENT

All participating youth provided their informed assent for their participation in the study. Parents of participating youth provided their permission for youth to participate in the study.

## Data Availability

The data that support the findings of this study are available on request from the corresponding author. The data are not publicly available due to privacy or ethical restrictions.
